# Author Correction: Profiling immunoglobulin repertoires across multiple human tissues using RNA sequencing

**DOI:** 10.1038/s41467-020-18509-2

**Published:** 2020-09-04

**Authors:** Igor Mandric, Jeremy Rotman, Harry Taegyun Yang, Nicolas Strauli, Dennis J. Montoya, William Van Der Wey, Jiem R. Ronas, Benjamin Statz, Douglas Yao, Velislava Petrova, Alex Zelikovsky, Roberto Spreafico, Sagiv Shifman, Noah Zaitlen, Maura Rossetti, K. Mark Ansel, Eleazar Eskin, Serghei Mangul

**Affiliations:** 1 Department of Computer Science, University of California, Los Angeles, 404 Westwood Plaza, Los Angeles, CA 90095 USA; 2 Department of Clinical Pharmacy, School of Pharmacy, University of Southern California, 1540 Alcazar Street, Los Angeles, CA 90033 USA; 3 Bioinformatics Interdepartmental Ph.D. Program, University of California, Los Angeles, 611 Charles E. Young Drive East, Los Angeles, CA 90095-1570 USA; 4 Biomedical Sciences Graduate Program, University of California, San Francisco, 1675 Owens Street, Suite 310, San Francisco, CA 94143-0523 USA; 5 Department of Molecular, Cell, and Developmental Biology, University of California, Los Angeles, 610 Charles E. Young Drive South, Los Angeles, CA 90095 USA; 6 Department of Microbiology, Immunology, and Molecular Genetics, University of California, Los Angeles, 609 Charles E. Young Drive East, Los Angeles, CA 90095 USA; 7 Program in Bioinformatics and Integrative Genomics, Harvard Medical School, 10 Shattuck Street, Suite 514, Boston, MA 02115 USA; 8 Wellcome Trust Sanger Institute, Wellcome Genome Campus, Hinxton, Cambridge, CB10 1SA UK; 9 Department of Computer Science, Georgia State University, 33 Gilmer Street SE, Atlanta, GA 30303 USA; 10 The Laboratory of Bioinformatics, I.M. Sechenov First Moscow State Medical University, Moscow, 119991 Russia; 11 Institute for Quantitative and Computational Biosciences, University of California, Los Angeles, 611 Charles E. Young Drive East, Los Angeles, CA 90095 USA; 12 Department of Genetics, The Institute of Life Sciences, The Hebrew University of Jerusalem, Jerusalem, 9190401 Israel; 13 Department of Medicine, University of California, San Francisco, 533 Parnassus Avenue, San Francisco, CA 94143 USA; 14 Immunogenetics Center, Department of Pathology and Laboratory Medicine, David Geffen School of Medicine, University of California, Los Angeles, 1000 Veteran Avenue, Los Angeles, CA 90095-1652 USA; 15 Sandler Asthma Basic Research Center, Department of Microbiology and Immunology, University of California, San Francisco, 513 Parnassus Avenue, San Francisco, CA 94143-0414 USA; 16 Department of Human Genetics, David Geffen School of Medicine at UCLA, 695 Charles E. Young Drive South, Box 708822, Los Angeles, CA 90095 USA; 17 Department of Computational Medicine, David Geffen School of Medicine at UCLA, 73-235 CHS, Los Angeles, CA 90095 USA

**Keywords:** Computational biology and bioinformatics, Adaptive immunity, Immunogenetics, B cells

Correction to: *Nature Communications* 10.1038/s41467-020-16857-7, published online 19 June 2020.

The original version of this article contained an error in the author affiliations.

Serghei Mangul was incorrectly associated with Quantitative and Computational Biology, University of Southern California, Los Angeles, CA 90089, USA.

This has now been corrected in both the PDF and HTML versions of the article.

