# Supplementary material for: Profiling immunoglobulin repertoires across multiple human tissues using RNA sequencing
Source: Nat Commun. 2020 Jun 19;11:3126. doi: 10.1038/s41467-020-16857-7 (PMC7305308; doi:10.1038/s41467-020-16857-7)
Supplement: Supplementary file 1 — Supplementary Information [file 41467_2020_16857_MOESM1_ESM.pdf]

Supplementary Information

Profiling immunoglobulin repertoires across multiple human tissues using RNA Sequencing

Mandric et al.

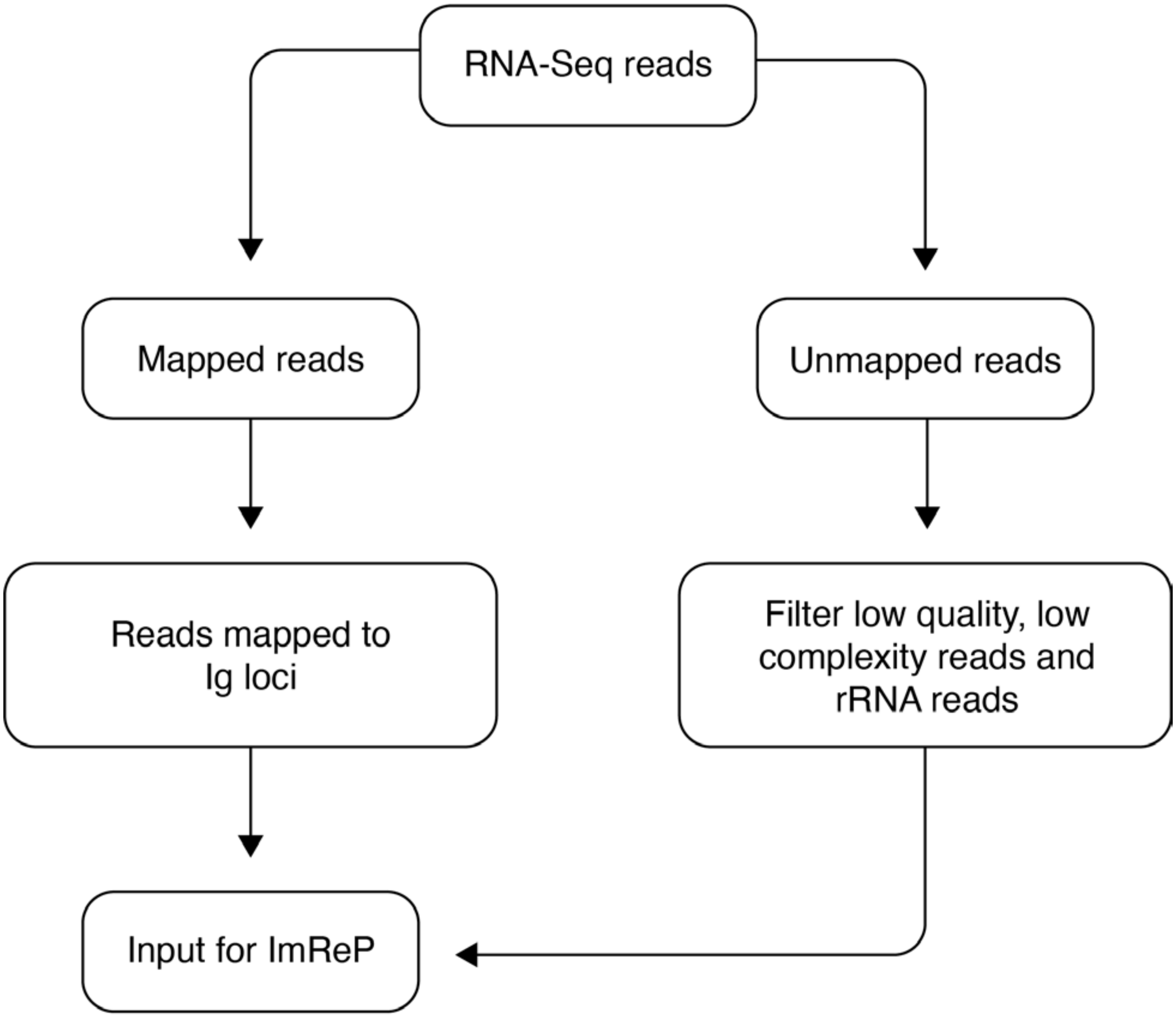

Supplementary Figure 1. Schematic visualizing protocol for selecting the candidate receptor-derived reads from RNA-Seq reads, which are the input for ImReP. Reads mapped to Ig loci are extracted from mapped reads. Low quality reads, low complexity reads, and reads from ribosomal DNA (rDNA) are filtered out from unmapped reads.

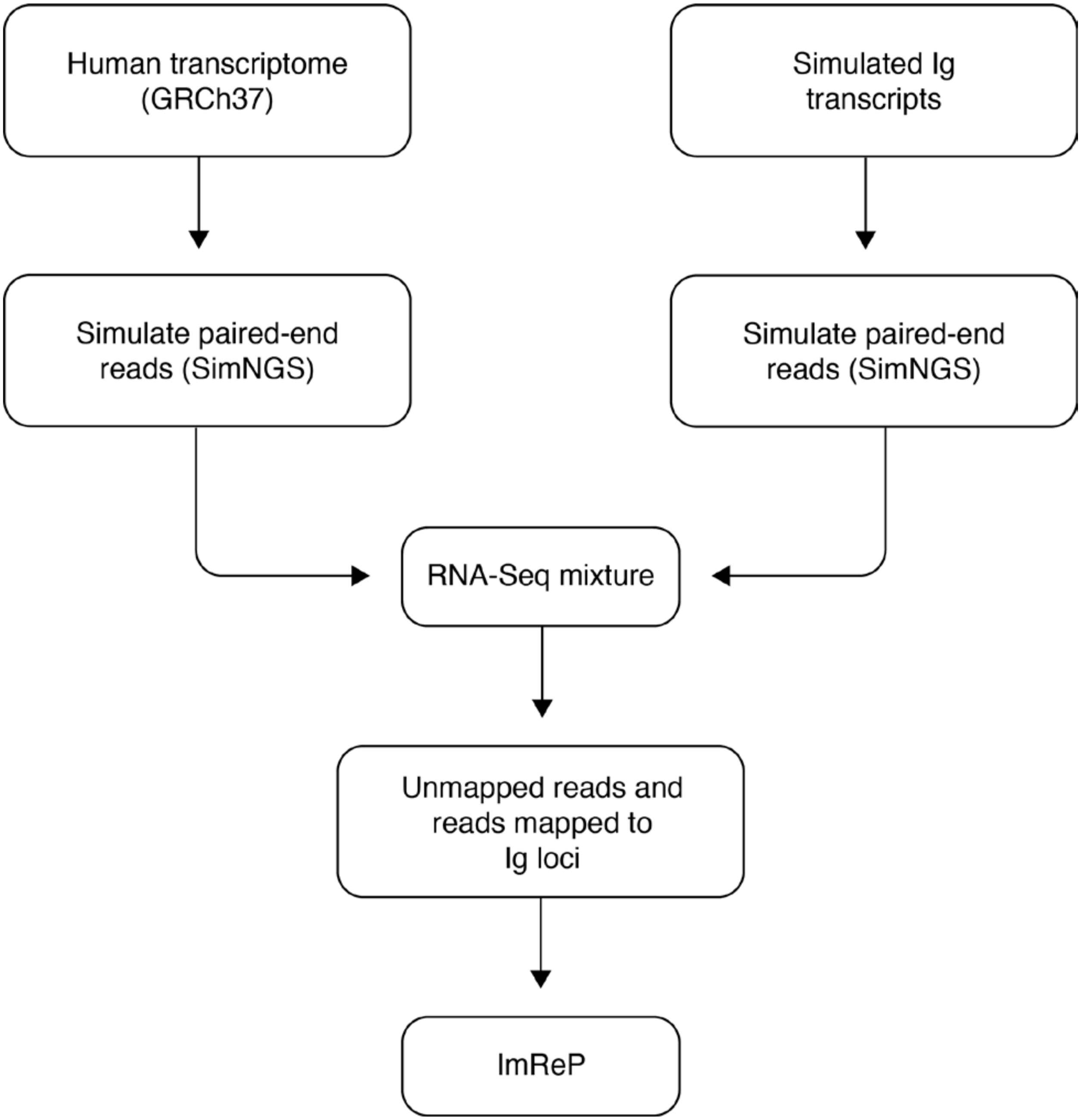

Supplementary Figure 2. Schematic visualization of the protocol for generating the mixture of transcriptomic and receptor-derived reads. Transcriptomic reads are simulated from the reference transcriptome. Receptor-derived reads are simulated from a mixture of Ig transcripts. Transcriptomics and receptor-derived reads are combined into RNA-Seq mixture.

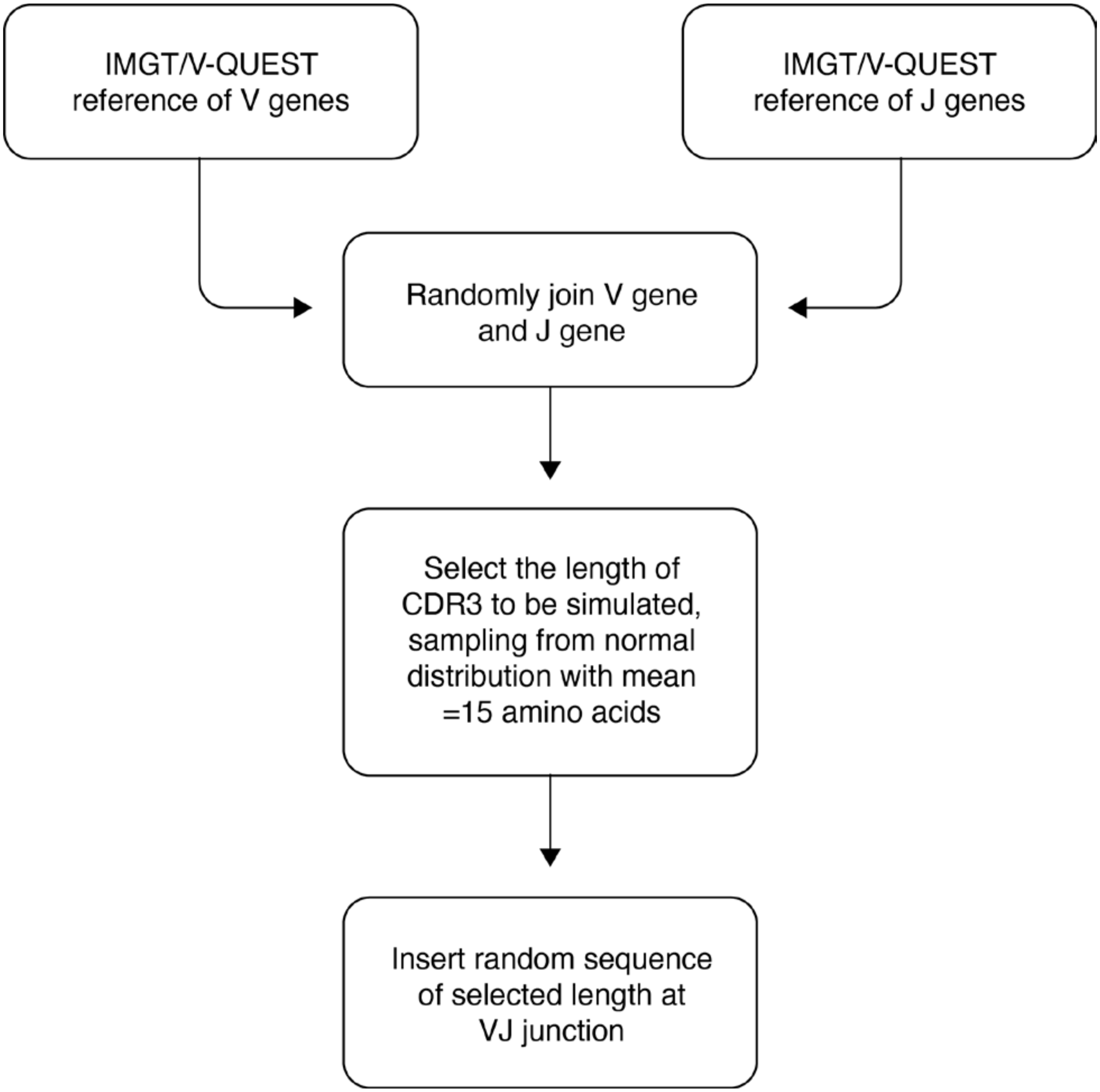

Supplementary Figure 3. Workflow for simulating Ig transcripts. A reference database containing V and J genes is used to simulate Ig transcripts. For each generation of a Ig transcript, one V-gene and one J-gene is randomly sampled from the database. Sample the length of the CDR3 region from a normal distribution with a mean value of 15 (amino acid). A random amino acid sequence of selected length is inserted at the V-J junction.

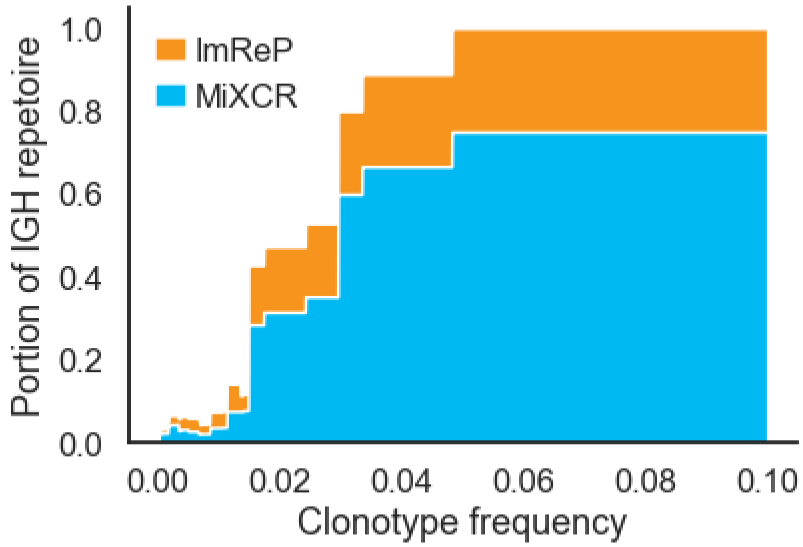

Supplementary Figure 4. Concordance of targeted BCR-Seq and non-specific RNA-Seq performed on 13 tumor biopsies from individuals diagnosed with Burkitt lymphoma. Area chart shows the proportion of the total IGH repertoire captured by ImRep (orange) and MiXCR (RNA-Seq mode) (blue), depending on the minimum BCR-seq-confirmed clonotypes frequency considered. The x-axis corresponds to BCR-seq-confirmed clonotypes frequency *Z*. The y-axis corresponds to the fraction of assembled IGH repertoires with clonotype abundances greater than *Z*. The total repertoire is defined as the total number of BCR-seq-confirmed clonotypes. Source data are provided as a Source Data file.

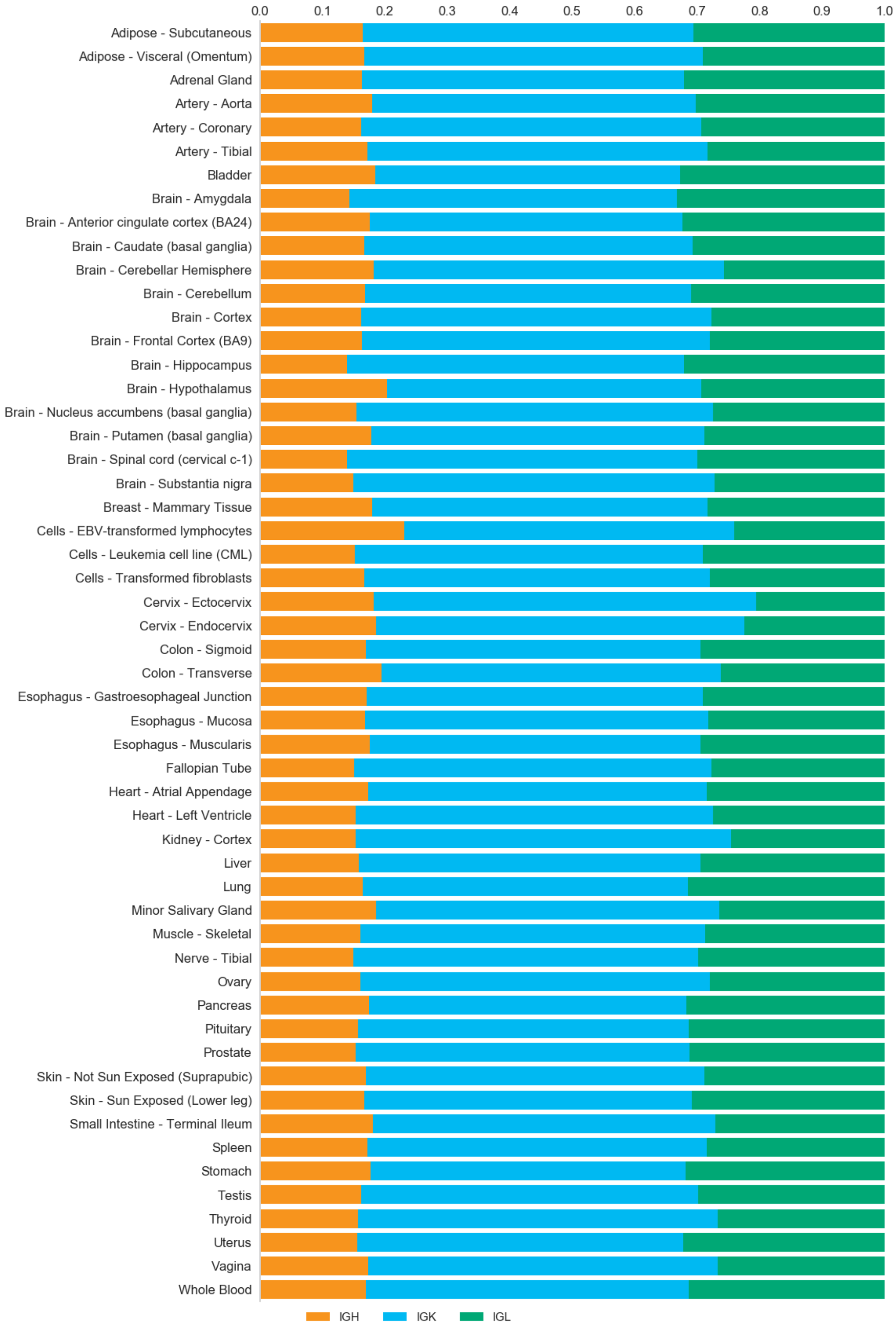

Supplementary Figure 5. The fraction of IGH, IGK, and IGL among the whole B cell population across 53 body sites. We have calculated the total number of Ig-derived reads by counting the reads derived from IGH, IGK, and IGL. The fraction of IGH, IGK, and IGL is calculated by dividing the IGH/IGK/IGL reads by the total number of Ig-derived reads. Source data are provided as a Source Data file.

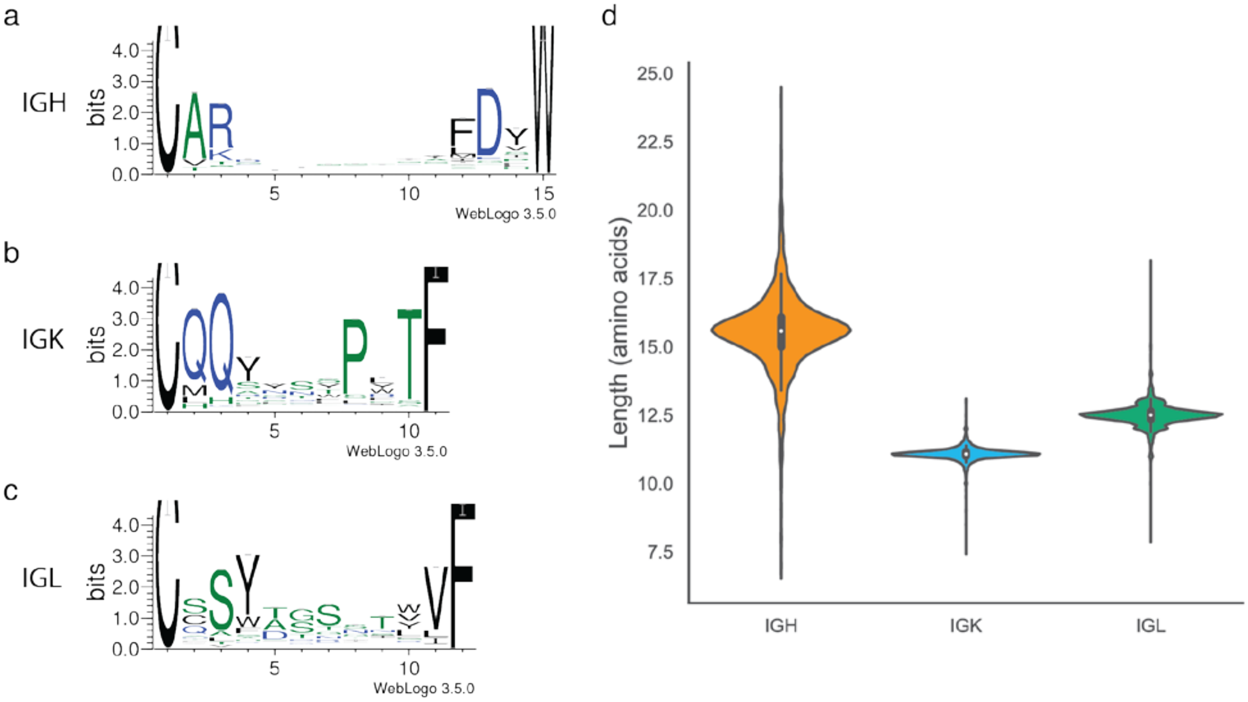

Supplementary Figure 6. The lengths and amino acid compositions of the assembled CDR3 sequences of immunoglobulin receptor chains. (a-c) Sequence logos (using WebLogo) of an (a) 15-amino-acid CDR3 sequence of IGH, (b) 11-amino-acid CDR3 of IGK, and (c) 12-amino-acid CDR3 sequence of IGL. The height of each amino acid within the stack indicates the relative frequency of this amino acid compared to other amino acids at the given position. (d) Distribution of CDR3 sequence length is estimated using *s* kernel density separately for each Ig chain, and visualized with a violin plot. Source data for (d) are provided as a Source Data file.

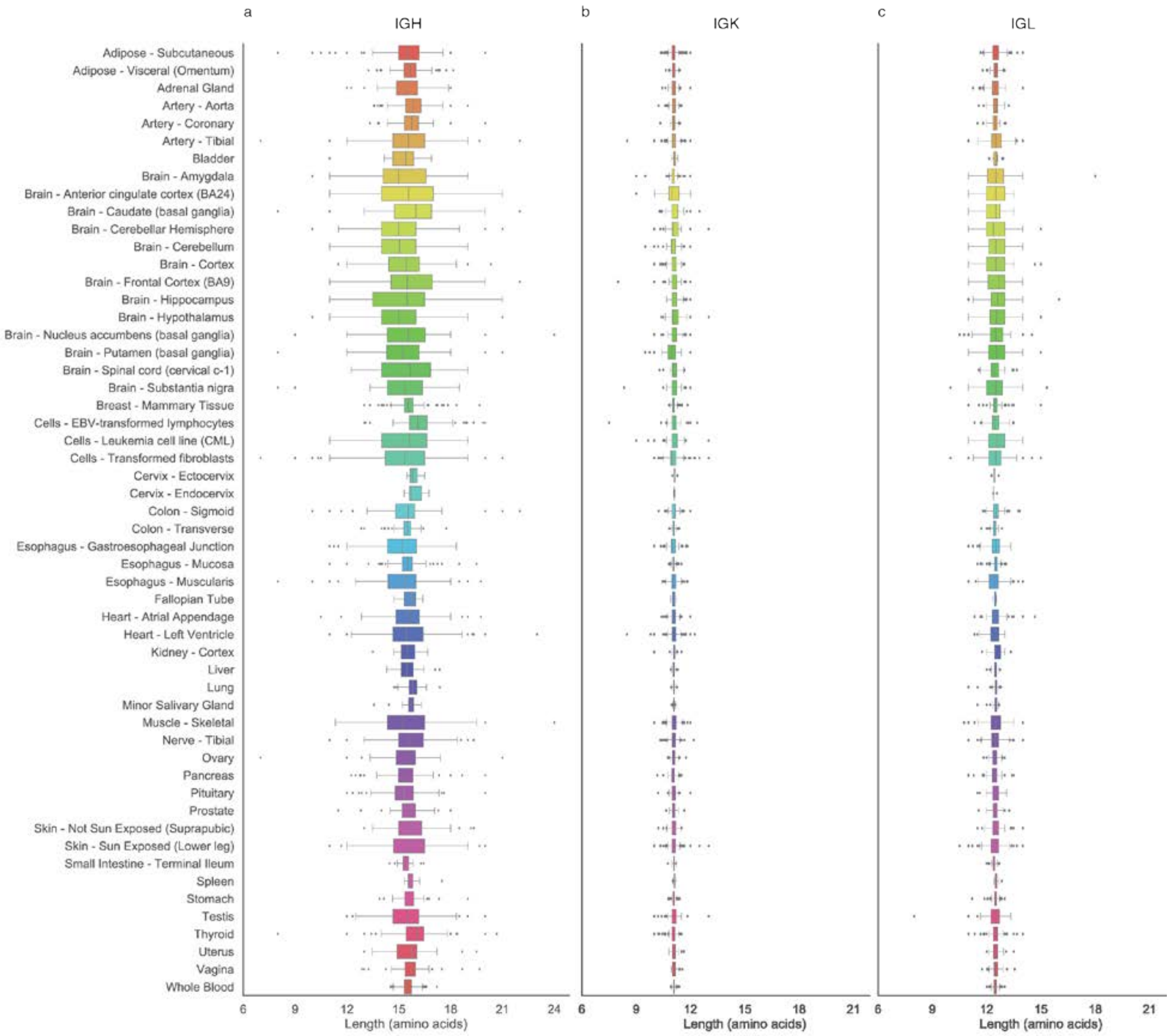

Supplementary Figure 7. The effect of tissue type on the length distribution of CDR3 sequences. Length distribution of amino acid sequences of the CDR3 region presented across 53 various body sites for (a) immunoglobulin heavy chain (IGH), (b) immunoglobulin kappa chain (IGK), and (c) immunoglobulin lambda chain (IGL). Source data are provided as a Source Data file.

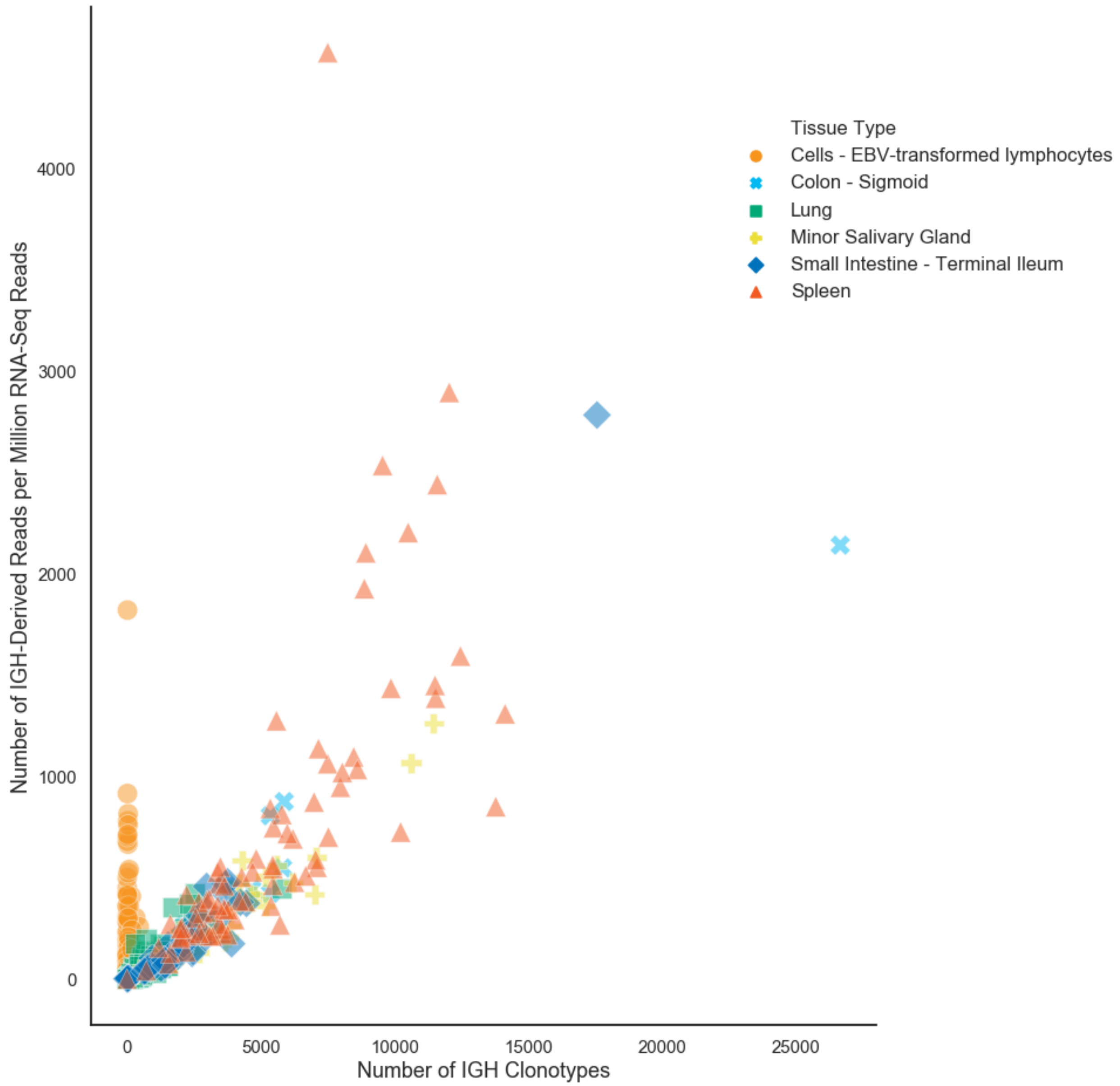

Supplementary Figure 8. Characteristics of immunoglobulin heavy chain clonotypes. Scatter plot depicting the number of IGH-derived reads per 1 million RNA-Seq reads (x-axis) and number of IGH-derived reads per 1 million RNA-Seq reads (y-axis). Source data are provided as a Source Data file.

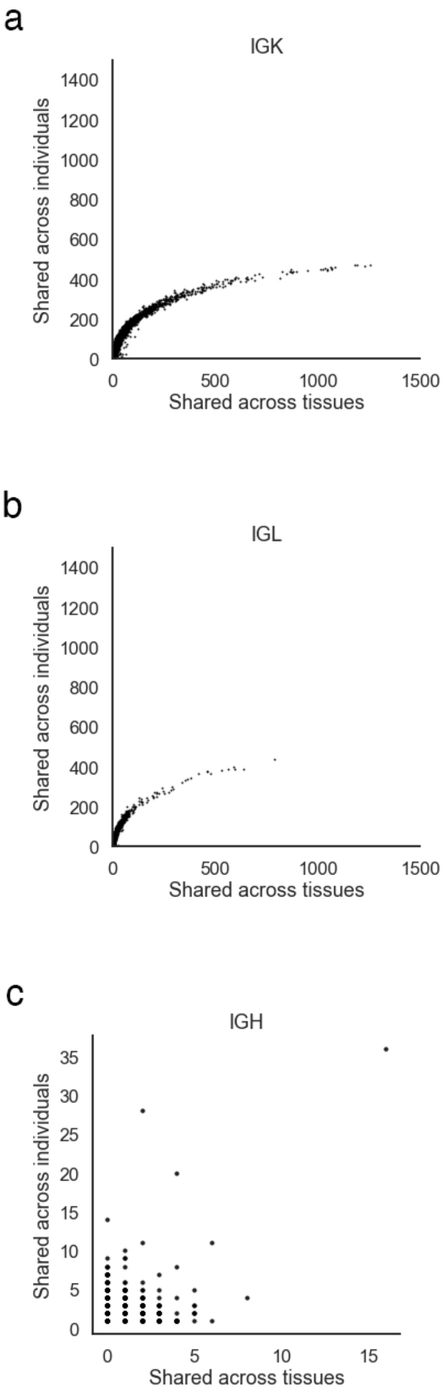

Supplementary Figure 9. Distribution of public *Ig* receptors across tissues. Scatter plot depicting the number of times receptor sequences were shared across tissues (x-axis) and across individuals (y-axis). (a) IGK (Pearson correlation: n = 459956, r = 0.78, p-value < $2x10^{-16}$), (b) IGL (Pearson correlation: n = 292680, r = 0.77, p-value < $2x10^{-16}$), and (c) IGH (Pearson correlation: n = 232898, r = 0.15, p-value < $2x10^{-16}$). Source data are provided as a Source Data file.

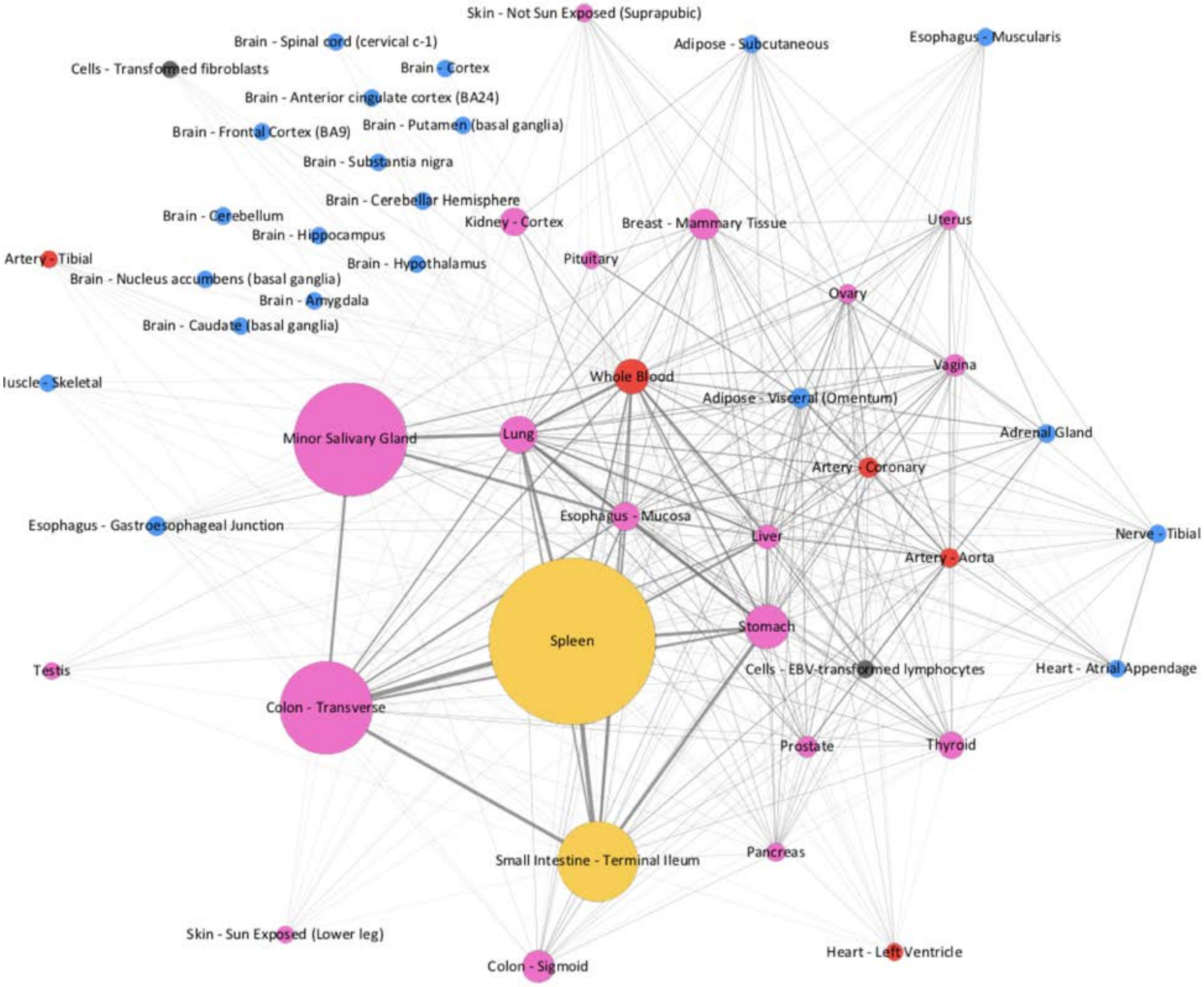

Supplementary Figure 10. The flow of IGK clonotypes across diverse human tissues presented as a network. The size of the node is proportional with the number of distinct clonotypes of the tissues. The thickness of edges are proportional to the compositional similarities between the tissues in terms of gain or loss of CDR3 sequences, as measured by beta diversity (Sørensen–Dice similarity index). Edges with beta diversity <.001 are not shown. Source data are provided as a Source Data file.

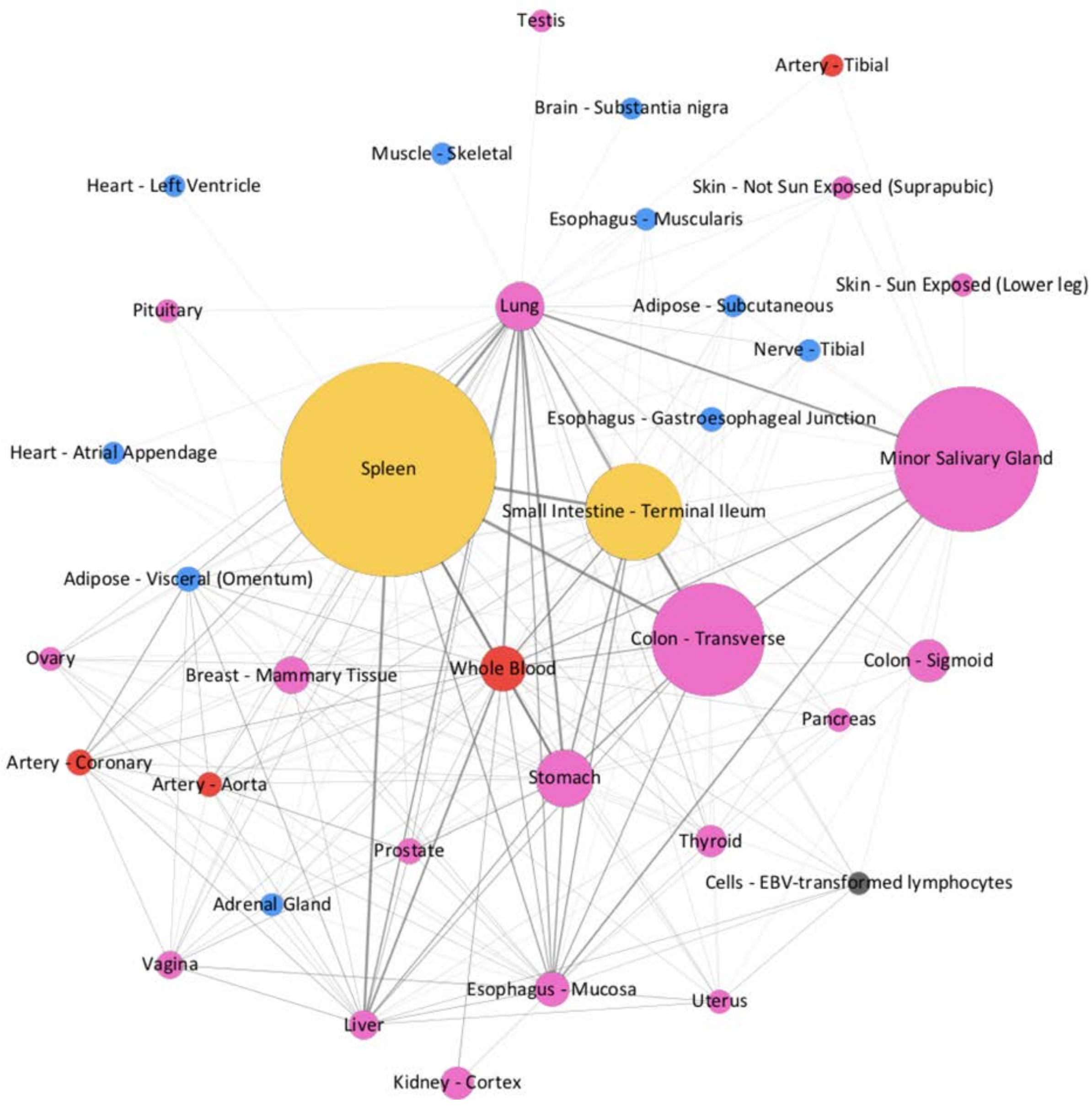

Supplementary Figure 11. The flow of IGL clonotypes across diverse human tissues presented as a network. The size of the node is proportional with the number of distinct clonotypes of the tissues. The thickness of edges are proportional to the compositional similarities between the tissues in terms of gain or loss of CDR3 sequences, as measured by beta diversity (Sørensen–Dice similarity index). Edges with beta diversity <.001 are not shown. Source data are provided as a Source Data file.

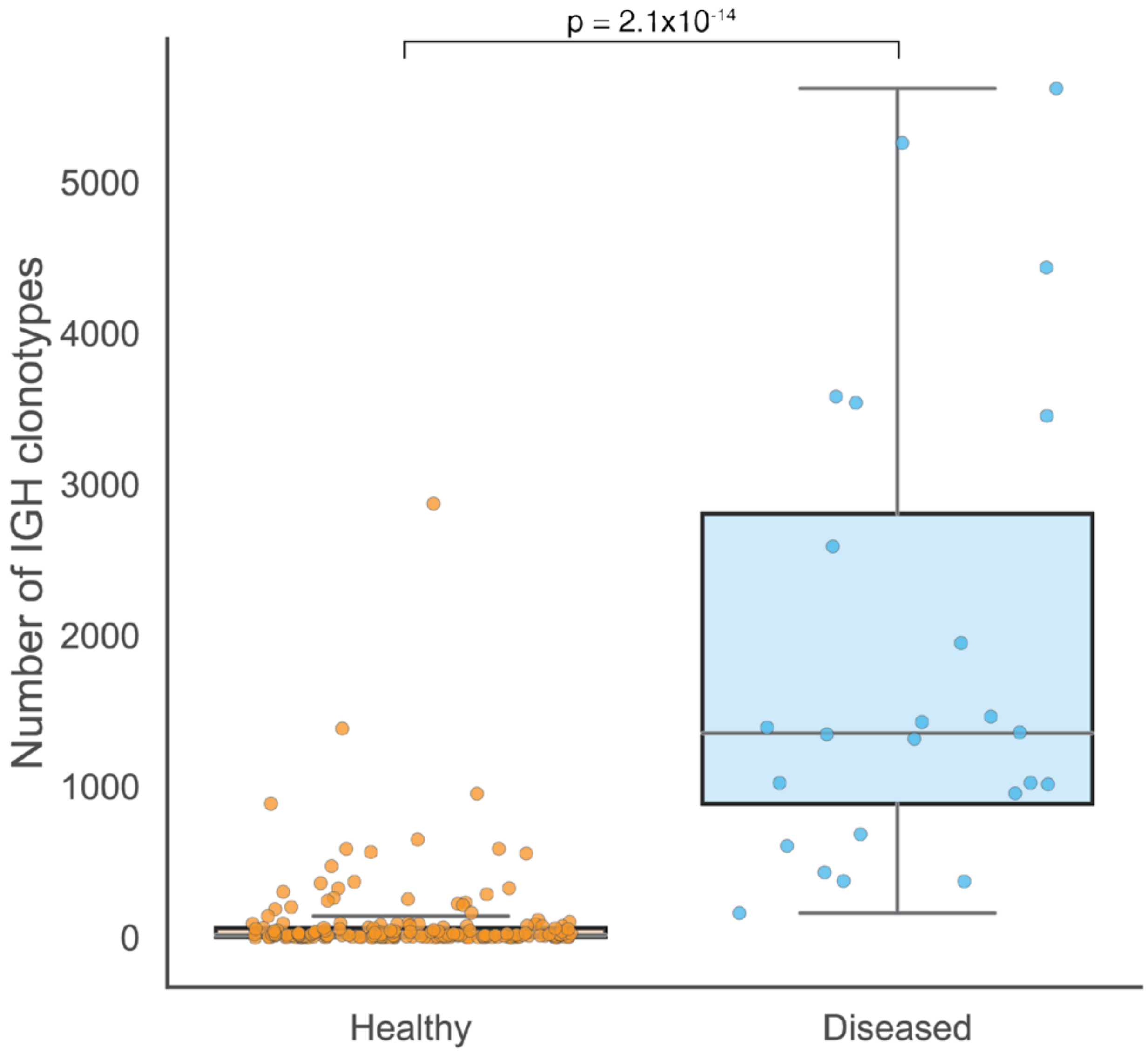

Supplementary Figure 12. The number of IGH clonotypes for healthy individuals (Healthy) and individuals diagnosed with Hashimoto's thyroiditis (Diseased). Pathologists' notes were used to annotate samples as either healthy (n = 180, min = 0, Q_1 = 5, median = 16, Q_3 = 61.75, max = 2872) or diseased (n = 24, min = 161, Q_1 = 886.5, median = 1351.5, Q_3 = 2805, max = 5621). Each box plot represents the median and interquartile range, with whiskers extending to 1.5 times the interquartile range. A significant increase in the number of distinct IGH clonotypes in samples with Hashimoto's thyroiditis (two-sided Mann-Whitney U test: U = 83, p-value = 2.1×10^{-14}) is observed. Source data are provided as a Source Data file.

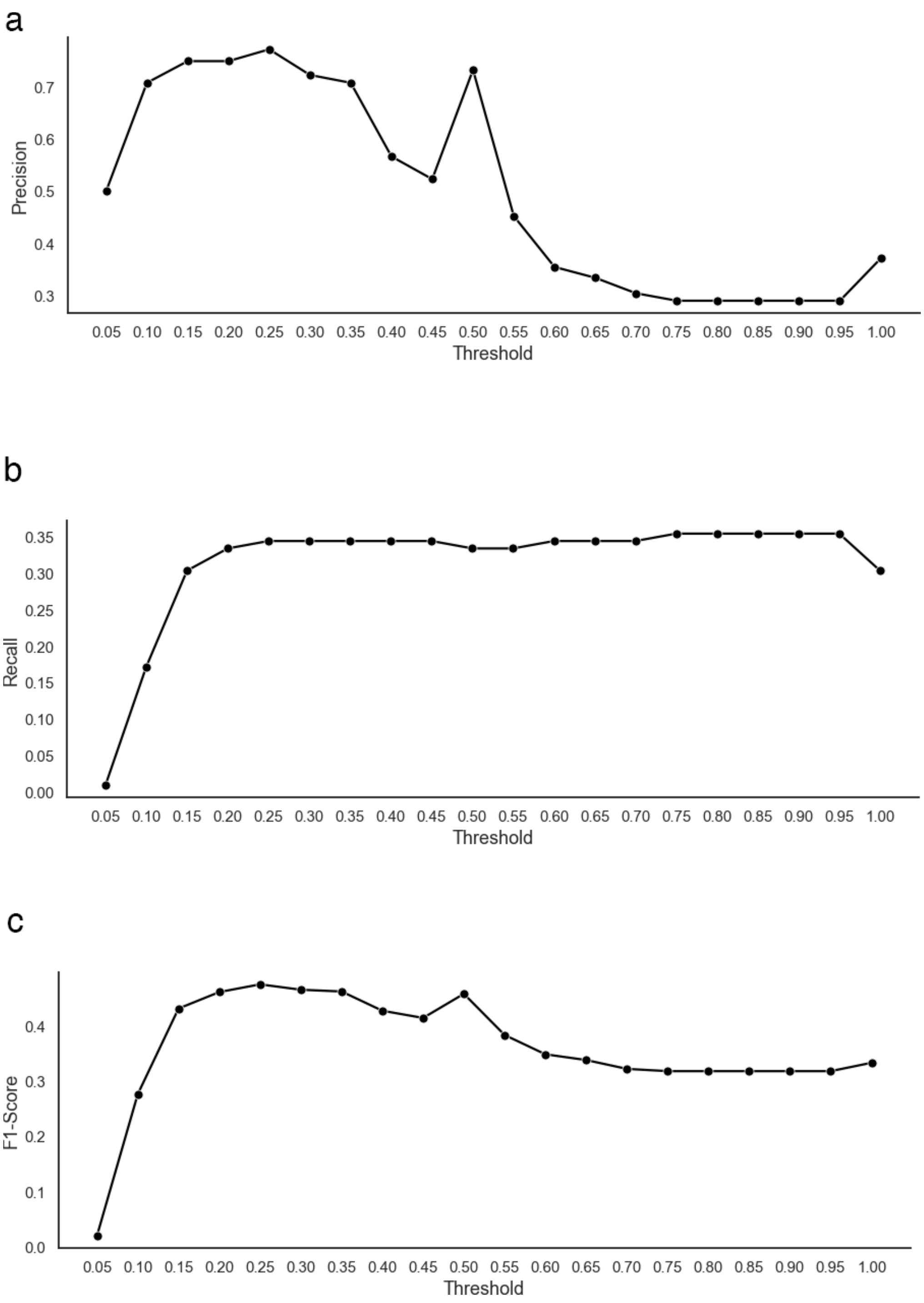

Supplementary Figure 13. Determining parameters for clustering using the CAST algorithm. The effect of the edit distance threshold used by the CAST clustering algorithm on precision (a), sensitivity (b), and F-score (c). Ig transcripts were simulated based on the random recombination of V and J gene segments (IMGT database) with non-template insertion at the recombination junction (see 'Validation based on simulated RNA-Seq data' in Methods). We simulated paired-end reads with a fixed length of 2x75bp, covering the Ig transcript with an average coverage rate of 8x. Source data are provided as a Source Data file.

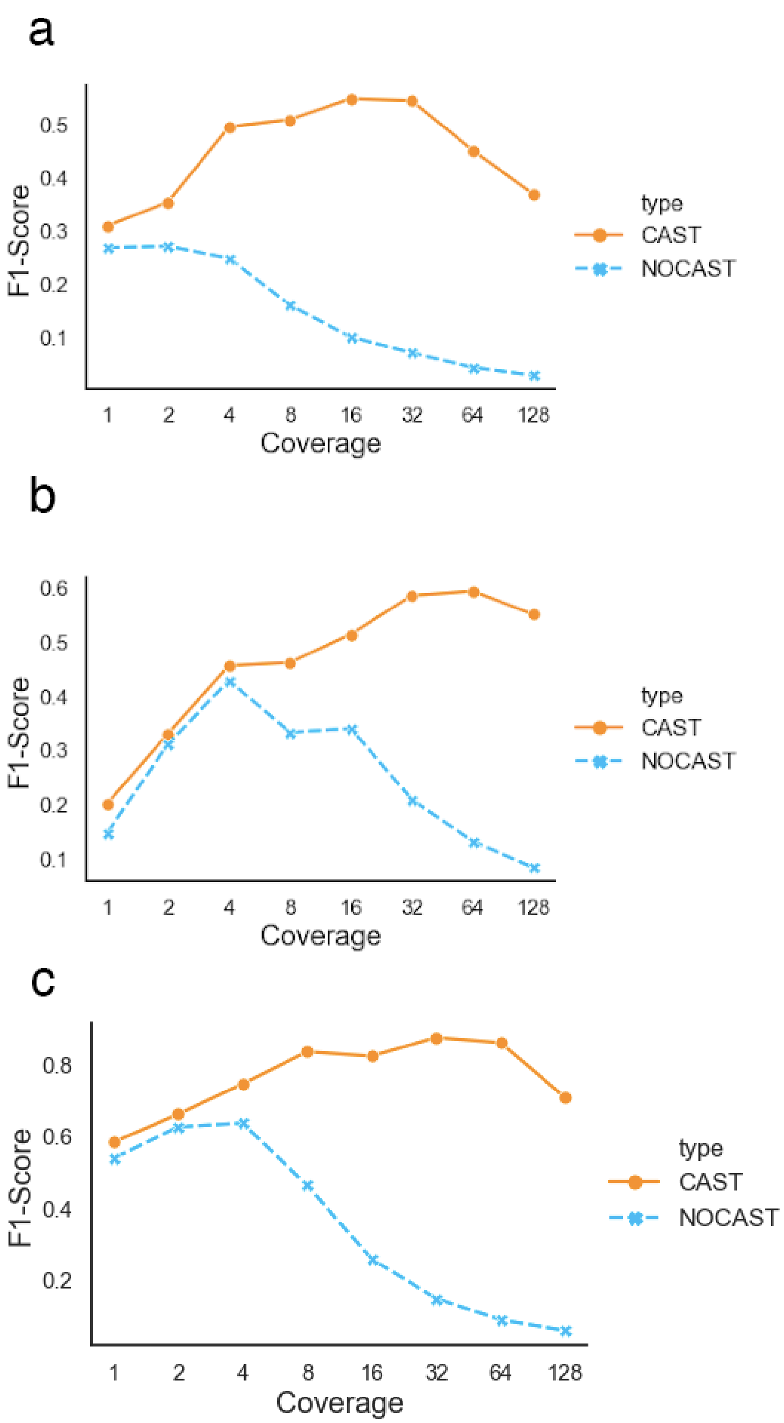

Supplementary Figure 14. The effect of the CAST clustering algorithm on the accuracy of assembled Ig clonotypes. Ig transcripts were simulated based on the random recombination of V and J gene segments (IMGT database), with non-template insertion at the recombination junction (see 'Validation based on simulated RNA-Seq data' in Methods). F-score rates for ImReP with CAST (blue), and ImReP without CAST (orange) on simulated data for immunoglobulin heavy (IGH) transcripts are reported for various reads lengths: (a) 50bp, (b) 75bp, and (c) 100bp—and per transcript coverages (1;2;4;8;16;32;64;128). Source data are provided as a Source Data file.

	Read length	Sensitivity	PPV	F-Score	TP	FP	TN
Overlap	50	21.0%	84.0%	33.6%	92	17	343
	75	25.0%	90.0%	39.1%	108	12	327
	100	26.0%	88.0%	40.1%	112	15	323
No overlap	50	5.0%	80.0%	9.4%	20	5	415
	75	21.0%	92.0%	34.2%	92	8	343
	100	26.0%	92.0%	40.5%	112	10	323

Supplementary Table 1. The effect of the overlap algorithm, which takes place during the second stage of ImReP, on the accuracy of assembled Ig clonotypes. IGH transcripts were obtained by targeted BCR-Seq (see 'Validation based on BCR-Seq-based IGH transcripts' in Methods). Reads were generated from BCR-Seq-based transcripts using simNGS (https://www.ebi.ac.uk/goldman-srv/simNGS/).

B cell signature		CD4+ Tcell Signature		CD8+ Tcell Signature	
HLA-DQA1	major histocompatibility complex, class II, DQ alpha 1	CD3D	CD3d molecule, delta (CD3-TCR complex)	LCK	lymphocyte-specific protein tyrosine kinase
HLA-DQA2	major histocompatibility complex, class II, DQ alpha 2	PTPRC	protein tyrosine phosphatase, receptor type, C	CD247	CD247 molecule
HLA-DMA	major histocompatibility complex, class II, DM alpha	LCK	lymphocyte-specific protein tyrosine kinase	CD3D	CD3d molecule, delta (CD3-TCR complex)
HLA-DOB	major histocompatibility complex, class II, DO beta	CD247	CD247 molecule	PIK3CD	phosphoinositide-3-kinase, catalytic, delta polypeptide

CXCR4	chemokine (C-X-C motif) receptor 4	PIK3CD	phosphoinositide-3-kinase, catalytic, delta polypeptide	CXCR4	chemokine (C-X-C motif) receptor 4
SELL	selectin L	CXCR4	chemokine (C-X-C motif) receptor 4	GZMA	granzyme A (granzyme 1, cytotoxic T-lymphocyte-associated serine esterase 3)
CD79A	CD79a molecule, immunoglobulin-associated alpha	IL7R	interleukin 7 receptor	CD48	CD48 molecule
ISG20	interferon stimulated exonuclease gene 20kDa	SELL	selectin L	IL7R	interleukin 7 receptor
MS4A1	membrane-spanning 4-domains, subfamily A, member 1	ICAM3	intercellular adhesion molecule 3	SELL	selectin L

CD37	CD37 molecule	IL10RA	interleukin 10 receptor, alpha	LTB	lymphotoxin beta (TNF superfamily, member 3)
CD48	CD48 molecule	CCR7	chemokine (C-C motif) receptor 7	CTSW	cathepsin W
LTB	lymphotoxin beta (TNF superfamily, member 3)	LTB	lymphotoxin beta (TNF superfamily, member 3)	HMHA1	histocompatibility (minor) HA-1
P2RX5	purinergic receptor P2X, ligand-gated ion channel, 5	CD48	CD48 molecule	CORO1 A	coronin, actin binding protein, 1A
LAPTM 5	lysosomal protein transmembrane 5	LAPTM 5	lysosomal protein transmembrane 5	KLRB1	killer cell lectin-like receptor subfamily B, member 1
PLAC8	placenta-specific 8	HMHA1	histocompatibility (minor) HA-1	NKG7	natural killer cell group 7 sequence
POU2A F1	POU class 2 associating factor 1	CORO1 A	coronin, actin binding protein, 1A	PLAC8	placenta-specific 8
TCL1A	T-cell	KLRB1	killer cell lectin-	DENND	DENN/MADD

	leukemia/lymphoma 1A		like receptor subfamily B, member 1	2D	domain containing 2D
FAIM3	Fas apoptotic inhibitory molecule 3	PLAC8	placenta-specific 8	FAIM3	Fas apoptotic inhibitory molecule 3
AL928768.3	lincRNA	DENND2D	DENN/MADD domain containing 2D	KLRC4	killer cell lectin-like receptor subfamily C, member 4
		FAIM3	Fas apoptotic inhibitory molecule 3		
		GMFG	glia maturation factor, gamma		

Supplementary Table 2. The top 25 genes with the highest proportional median value for CD19+ B cells, CD4+ T cells, and CD8+ T cells. Each gene signature shown are the genes from the top 25 proportional median genes that overlap in the GTEx dataset. Therefore, the lists range from 19 to 21 genes.
