# Supplementary material for: Profiling immunoglobulin repertoires across multiple human tissues using RNA sequencing
Source: Nat Commun. 2020 Jun 19;11:3126. doi: 10.1038/s41467-020-16857-7 (PMC7305308; doi:10.1038/s41467-020-16857-7)
Supplement: Supplementary file 3 — Reporting Summary [file 41467_2020_16857_MOESM3_ESM.pdf]

nature research

Double-blind peer review submissions: write DBPR and your manuscript number here instead of author names.

Corresponding author(s): Serghei Mangul

Last updated by author(s): Apr 20, 2020

Reporting Summary

Nature Research wishes to improve the reproducibility of the work that we publish. This form provides structure for consistency and transparency in reporting. For further information on Nature Research policies, see Authors & Referees and the Editorial Policy Checklist .

Statistics

For all statistical analyses, confirm that the following items are present in the figure legend, table legend, main text, or Methods section.

n/a	Confirmed	
[ ]	[x]	The exact sample size (*n*) for each experimental group/condition, given as a discrete number and unit of measurement
[ ]	[x]	A statement on whether measurements were taken from distinct samples or whether the same sample was measured repeatedly
[ ]	[x]	The statistical test(s) used AND whether they are one- or two-sided<br>*Only common tests should be described solely by name; describe more complex techniques in the Methods section.*
[x]	[ ]	A description of all covariates tested
[x]	[ ]	A description of any assumptions or corrections, such as tests of normality and adjustment for multiple comparisons
[x]	[ ]	A full description of the statistical parameters including central tendency (e.g. means) or other basic estimates (e.g. regression coefficient) AND variation (e.g. standard deviation) or associated estimates of uncertainty (e.g. confidence intervals)
[x]	[ ]	For null hypothesis testing, the test statistic (e.g. *F*, *t*, *r*) with confidence intervals, effect sizes, degrees of freedom and *P* value noted<br>*Give P values as exact values whenever suitable.*
[x]	[ ]	For Bayesian analysis, information on the choice of priors and Markov chain Monte Carlo settings
[x]	[ ]	For hierarchical and complex designs, identification of the appropriate level for tests and full reporting of outcomes
[x]	[ ]	Estimates of effect sizes (e.g. Cohen's *d*, Pearson's *r*), indicating how they were calculated

Our web collection on statistics for biologists contains articles on many of the points above.

Software and code

Policy information about availability of computer code

Data collection	No data was collected for this study.
Data analysis	All code required to produce the figures and analysis performed in this paper are freely available at https://github.com/Mangul-Lab-USC/ImReP_publication. We have used the following bioinformatics tools: Igblast (version 1.6.1), LymAnalyzer (version 1.2.2), simNGS (version 1.6), simLibrary (version 1.3), IMSEQ (version 1.1.0) , MiXCR (version 2.2).

For manuscripts utilizing custom algorithms or software that are central to the research but not yet described in published literature, software must be made available to editors/reviewers. We strongly encourage code deposition in a community repository (e.g. GitHub). See the Nature Research guidelines for submitting code & software for further information.

Data

Policy information about availability of data

All manuscripts must include a data availability statement. This statement should provide the following information, where applicable:

All RNA-Seq data discussed in this paper is available as part of the Genotype-Tissue Expression (GTEx) Project under phs000424.v8.p2 accession number in the database of Genotypes and Phenotypes (dbGaP). The targeted BCR-Seq assemblies used for validation of ImReP method are available at the adaptive biosystems webpage (https://clients.adaptivebiotech.com/pub/lombardo-2017-bloodadvances).
RNA-Seq samples used for validation of ImReP are available under SRP099346 accession number in the Sequence Read Archive. Raw sequence reads of targeted BCR-Seq data are available under the EGAN00001419382 accession number in the European Genome-Phenome Archive. All data required to produce the figures and analysis performed in this paper are freely available at https://github.com/Mangul-Lab-USC/ImReP_publication.
This includes Figures 2a-f, 3, 4a, 4c, 5a-b, 6a-b, S4, S5, S6a-d, S7, S8, S9a-c, S10, S11, S12, S13a-c, and S14a-c.

Field-specific reporting

Please select the one below that is the best fit for your research. If you are not sure, read the appropriate sections before making your selection.

[x] Life sciences   [ ] Behavioural & social sciences   [ ] Ecological, evolutionary & environmental sciences

Life sciences study design

All studies must disclose on these points even when the disclosure is negative.

Sample size	This study did not generate the data and thus sample size determination was impossible
Data exclusions	No data were excluded from this study
Replication	Replication was not possible for the study since no data was generated instead this study was using public data, where replication was not available.
Randomization	Randomization was not possible for the study since no data was generated instead this study was using public data, where randomization was not available.
Blinding	Blinding was not possible for the study since no data was generated instead this study was using public data, where blinding was not available.

Reporting for specific materials, systems and methods

We require information from authors about some types of materials, experimental systems and methods used in many studies. Here, indicate whether each material, system or method listed is relevant to your study. If you are not sure if a list item applies to your research, read the appropriate section before selecting a response.

Materials & experimental systems

n/a	Involved in the study
☒	☐ Antibodies
☒	☐ Eukaryotic cell lines
☒	☐ Palaeontology
☒	☐ Animals and other organisms
☒	☐ Human research participants
☒	☐ Clinical data

Methods

n/a	Involved in the study
☒	☐ ChIP-seq
☒	☐ Flow cytometry
☒	☐ MRI-based neuroimaging

ChIP-seq

Data deposition

☐ Confirm that both raw and final processed data have been deposited in a public database such as GEO.

☐ Confirm that you have deposited or provided access to graph files (e.g. BED files) for the called peaks.

Data access links<br>*May remain private before publication.*	N/A
Files in database submission	N/A
Genome browser session<br>(e.g. UCSC)	N/A

Methodology

Replicates	N/A
Sequencing depth	N/A
Antibodies	N/A
Peak calling parameters	N/A
Data quality	N/A
Software	N/A

Flow Cytometry

Plots

Confirm that:

- [ ] The axis labels state the marker and fluorochrome used (e.g. CD4-FITC).
- [ ] The axis scales are clearly visible. Include numbers along axes only for bottom left plot of group (a 'group' is an analysis of identical markers).
- [ ] All plots are contour plots with outliers or pseudocolor plots.
- [ ] A numerical value for number of cells or percentage (with statistics) is provided.

Methodology

Sample preparation	N/A
Instrument	N/A
Software	N/A
Cell population abundance	N/A
Gating strategy	N/A

- [ ] Tick this box to confirm that a figure exemplifying the gating strategy is provided in the Supplementary Information.

Magnetic resonance imaging

Experimental design

Design type	N/A
Design specifications	N/A
Behavioral performance measures	N/A

Acquisition

Imaging type(s)	N/A
Field strength	N/A
Sequence & imaging parameters	N/A
Area of acquisition	N/A

Diffusion MRI [ ] Used [x] Not used

Preprocessing

Preprocessing software	N/A
Normalization	N/A
Normalization template	N/A
Noise and artifact removal	N/A
Volume censoring	N/A

Statistical modeling & inference

Model type and settings	N/A
Effect(s) tested	N/A

Specify type of analysis: [ ] Whole brain [ ] ROI-based [ ] Both

Statistic type for inference (See Eklund et al. 2016)	N/A
Correction	N/A

Models & analysis

n/a	Involved in the study
☒	☐ Functional and/or effective connectivity
☒	☐ Graph analysis
☒	☐ Multivariate modeling or predictive analysis
