# Supplementary material for: Profiling immunoglobulin repertoires across multiple human tissues using RNA sequencing
Source: Nat Commun. 2020 Jun 19;11:3126. doi: 10.1038/s41467-020-16857-7 (PMC7305308; doi:10.1038/s41467-020-16857-7)
Supplement: Supplementary file 4 — Description of Additional Supplementary Files [file 41467_2020_16857_MOESM4_ESM.pdf]

Description of Additional Supplementary Files

File Name: Supplementary Data 1
Description: Data Overview. Characteristics of 8,555 samples across 544 individuals from 53 body sites obtained from the Genotype-Tissue Expression study (GTEx v6). The second column reports the tissue type based on the relationship to the immune system. The tissues inside each tissue type group are sorted based on the number of CDR3 sequences. (3) The histological type of the body site. (4) Median number of 76x2 bp paired-end reads per sample. (5) Number of RNA-Seq samples available via GTEx. Results for (7-40) are presented individually for immunoglobulin heavy chain (IGH), immunoglobulin kappa chain (IGK, immunoglobulin lambda chain (IGK), T cell receptor alpha chain (TCRA), T cell receptor beta chain (TCRB), T cell receptor delta chain (TCRD), and T cell receptor gamma chain (TCRG). (7-8) Median relative abundance of B or T cells within each tissue. (10-16) Median number of distinct CDR3 (clonotypes) per tissue. (18-24) Median number of distinct clonotypes (CDR3) per 1 million RNA-Seq reads (CPM). (34-40) We used per sample alpha diversity (Shannon entropy) to estimate the diversity of the immune repertoire.

File Name: Supplementary Data 2
Description: Concordance of targeted BCR-Seq and non-specific RNA-seq. We used 13 tumor biopsies from individuals diagnosed with Burkitt lymphoma that were sequenced both by BCR-Seq and RNA-Seq. For each biopsy (row), we report the major and minor clonotype frequencies given by targeted BCR-Seq (2 and 3) and derived from non-specific RNA-Seq using ImRep (6 and 7) and MiXCR (8 and 9). The portion of the IGH repertoire captured is reported for both ImRep (4) and MiXCR (5). In addition, we report the total number of clonotypes found through each method: (10) BCR-seq, (11) ImRep, and (12) MiXCR.

File Name: Supplementary Data 3
Description: The adjusted clonotypic richness of B cells, calculated as the number of distinct amino acid sequences of CDR3 per one million RNA-Seq reads (CPM) and normalized by the proportion of the B cell in the sample. We used SaVant, a transcriptome-based computational method, to infer the relative abundance of B cells within each tissue sample based on cell-specific gene signatures (independent of Ig transcripts).

File Name: Supplementary Data 4
Description: GTEx sample metadata. All data used can be found on the NCBI Sequence Read Archive (SRA). Here we have compiled all of the metadata for each GTEx sample used in this paper.
